# Autonomic nervous system development-related signature as a novel predictive biomarker for immunotherapy in pan-cancers

**DOI:** 10.3389/fimmu.2025.1611890

**Published:** 2025-07-23

**Authors:** Cunen Wu, Weiwei Xue, Yuwen Zhuang, Dayue Darrel Duan, Zhou Zhou, Xiaoxiao Wang, Zhenfeng Wu, Jin-yong Zhou, Xiangkun Huan, Ruiping Wang, Haibo Cheng

**Affiliations:** ^1^ Department of Oncology, Affiliated Hospital of Nanjing University of Chinese Medicine, Jiangsu Province Hospital of Chinese Medicine, Nanjing, Jiangsu, China; ^2^ Jiangsu Collaborative Innovation Center of Traditional Chinese Medicine Prevention and Treatment of Tumor, Nanjing, Jiangsu, China; ^3^ Department of Surgery/Division of General Surgery, Beth Israel Deaconess Medical Center, Boston, MA, United States; ^4^ Harvard Medical School, Boston, MA, United States; ^5^ The Academy of Phenomics of TCM, and School of Integrated Medicine, Nanjing University of Chinese Medicine, Nanjing, Jiangsu, China; ^6^ Department of Pharmacology, University of Nevada Reno School of Medicine, Reno, NV, United States; ^7^ Oncology Department of Integrated Chinese and Western Medicine, First Affiliated Hospital of Anhui Medical University, Hefei, Anhui, China; ^8^ Department of GCP Research Center, Affiliated Hospital of Nanjing University of Chinese Medicine, Jiangsu Province Hospital of Chinese Medicine, Nanjing, Jiangsu, China; ^9^ Department of Surgical Oncology, Affiliated Hospital of Nanjing University of Chinese Medicine, Jiangsu Province Hospital of Chinese Medicine, Nanjing, Jiangsu, China; ^10^ Department of Key Laboratory, Affiliated Hospital of Nanjing University of Chinese Medicine, Jiangsu Province Hospital of Chinese Medicine, Nanjing, Jiangsu, China

**Keywords:** immunotherapy, predicting tool, ANSD related signature, machine learning, single-cell RNA sequencing, pan-cancer

## Abstract

**Background:**

Immunotherapy has revolutionized cancer treatment. However, its clinical application remains limited. There is an urgent need for new predictive and prognostic biomarkers that can identify more patients with objective and durable responses and thus, improve the accuracy of prognosis.

**Methods:**

A predictive model for immunotherapy was developed using 34 single-cell RNA sequencing (scRNA-Seq) datasets from various cancer types and eight bulk RNA-Seq datasets from immune checkpoint inhibitor (ICI) cohorts. Seven machine learning (ML) methods were applied to identify vital genes associated with both cancer and immune characteristics. Differentially expressed genes (DEGs) were validated using RT-PCR and immunohistochemical (IHC) analyses of clinical samples.

**Results:**

Analysis of scRNA-seq datasets and autonomic nervous system development (ANSD) scores revealed 20 genes comprising a novel ANSD-related differential signature (ANSDR.Sig). A pan-cancer predictive model for ICI prognosis based on ANSDR.Sig was constructed, with the random forest algorithm yielding the most robust performance. Further screening using five ML methods on the ICI RNA-seq datasets identified 18 key genes, forming the Hub-ANSDR.Sig. Regulatory network analysis revealed diversified molecular interactions between Hub-ANSDR.Sig genes, transcription factors, and miRNAs. Hub-ANSDR.Sig was strongly associated with immune cell infiltration, microsatellite instability (MSI), and overall survival (OS) across various cancer types. In gastric cancer (GC), its role in immune dysfunction, tumor mutational burden (TMB), MSI, mutation frequency, immune infiltration, cell–cell communication, and developmental trajectories was confirmed. Moreover, several Hub-ANSDR.Sig genes were differentially expressed in GC compared to normal tissue and were enriched in immunotherapy-sensitive GC samples relative to resistant ones.

**Conclusion:**

Our results offer novel insights into predicting immunotherapy efficacy using ANSD-related signature, with the goal of improving clinical strategies and expanding potential indications. This approach also aims to develop more accurate prediction models and therapeutic interventions, thereby helping more patients benefit from immunotherapy.

## Introduction

Cancer ranks as the second most common cause of death globally, with a rising incidence that continues to burden health systems worldwide (1). In addition to traditional therapies, immunotherapy—which aims to take advantage of the body’s own immune system to combat cancer —has revolutionized the field of cancer treatment (2). Immunotherapy has been extensively used in clinical treatment across various cancer types due to its advantages in achieving durable disease control. Among these approaches, immune checkpoint inhibitors (ICIs) represent one of the most universal options in immunotherapy (3). However, only a subset of patients respond positively to ICIs, and existing biomarkers fail to accurately predict outcomes (4), underscoring the urgent need for effective predictive tools to guide more precise immunotherapy strategies.

The autonomic nervous system (ANS), comprising the sympathetic and parasympathetic branches, operates independently of conscious control and plays a synergistic role in regulating visceral responses to environmental changes (5, 6). The ANS participates in multiple physiological and pathological processes involving almost all organ systems, which highlights its potential therapeutic relevance (7). Recent studies have revealed that sympathetic nervous system activation by environmental eustress can modulate the β-adrenergic receptor/CCL2 axis, thereby sensitizing tumors to immunotherapy in liver cancer (8). In addition, sympathetic activity associated with stress has been shown to induce T cell exhaustion via the β1-adrenergic receptor (ADRB1), and deletion of ADRB1 enhances immunotherapy efficacy in both melanoma and pancreatic cancer (9). Given the well-established role of the immune microenvironment, further exploration into the role of the ANS in cancer immunotherapy is warranted.

Recent advancements in single-cell RNA sequencing (scRNA-seq) have shed light on the cellular mechanisms underlying cancer-immune interactions (10). At the same time, machine learning (ML) has emerged as a powerful tool in cancer diagnosis and therapy, capable of enhancing predictive performance by automatically selecting relevant variables and modeling complex nonlinear relationships (4). ML-assisted identification of cancer immunophenotypes holds great promise for forecasting treatment responses and optimizing clinical decision-making (11).

**Figure 1:**
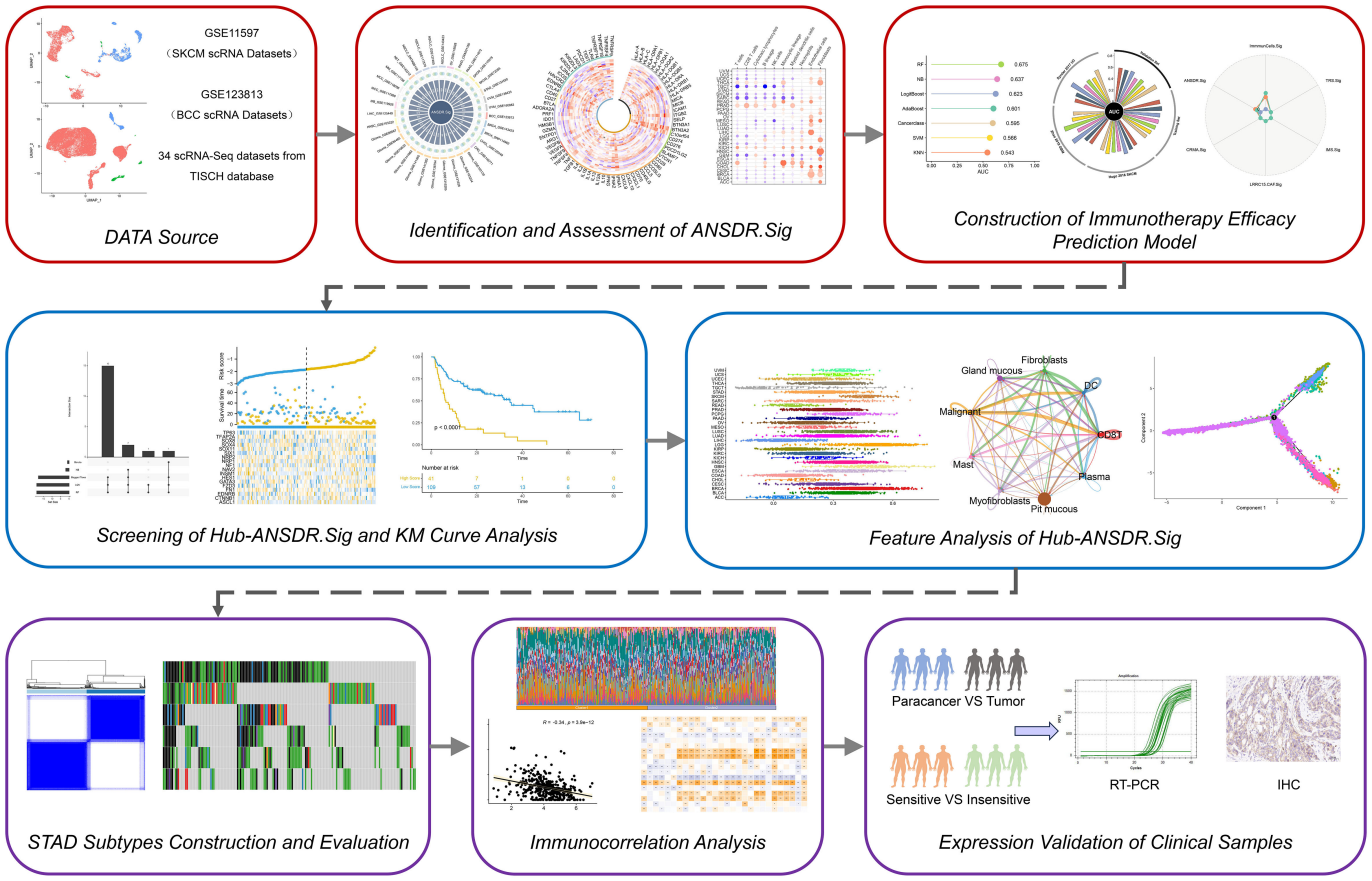
Graphic overview of research design. Thirty-four scRNA-seq datasets were obtained from the TISCH database. ANSDR.Sig was derived by identifying differentially expressed genes and evaluating their association with the ANSD score. Pan-cancer ICI prediction models were constructed based on ANSDR.Sig using seven ML methods. Hub-ANSDR.Sig was generated by assessing the relationship between ANSDR.Sig and immunotherapy efficacy via five ML algorithms. Molecular interactions within Hub-ANSDR.Sig were further explored through mRNA–transcription factor (TF) and mRNA-miRNA) networks. In addition, correlations between Hub-ANSDR.Sig and immune cell infiltration, MSI, OS across cancer types were analyzed. The role of Hub-ANSDR.Sig in dysfunction, TMB, MSI, mutation frequency, immune infiltration, cell–cell communication, and developmental trajectories in GC was also investigated. Clinical samples were collected to examine differential expression of Hub-ANSDR.Sig.

In this study, as shown in **Figure 1**, we identified a gene set, ANSDR.Sig, by integrating differentially expressed genes with autonomic nervous system development (ANSD) scores across 34 scRNA-seq datasets. Using seven ML algorithms, we developed pan-cancer ICI prediction models based on ANSDR.Sig. We further examined the association between ANSDR.Sig and immunotherapy efficacy using five ML methods, identifying 18 key genes that constituted a refined gene set, Hub-ANSDR.Sig. To elucidate regulatory mechanisms, we analyzed mRNA–transcription factor (TF) and mRNA-miRNA interactions within the Hub-ANSDR.Sig network. We also investigated the relationship between Hub-ANSDR.Sig and immune cell infiltration, MSI, and OS across different cancer types. In gastric cancer (GC), we explored the role of Hub-ANSDR.Sig in immune dysfunction, tumor mutational burden (TMB), MSI, mutation frequency, immune infiltration, cell–cell communication, and developmental trajectories. To enhance clinical significance, we analyzed paired GC patient samples as well as tissues stratified by immunotherapy sensitivity to evaluate differential expression of Hub-ANSDR.Sig genes. Collectively, our findings demonstrate the impact of ANSD -related signatures on key cancer–immunity processes and aim to contribute to the development of more accurate prediction models and effective immunotherapeutic interventions to benefit a broader patient population.

## Methods

### Analysis of scRNA-seq data

The integration and analysis of scRNA-seq data were implemented using the R package Seurat v4.0.6. Quality control of double cells was conducted using the R package Scrublet. Cells with less than 300 genes and those with more than 20% mitochondrial gene reads were removed during quality control. The data for each sample were normalized using by principal component analysis (PCA), and batch effects between samples were eliminated using the R package Harmony. Dimensionality reduction and visualization of scRNA-seq data were performed using the Uniform Manifold Approximation and Projection (UMAP) algorithm. The “FindMarkers” function was used to identify differentially expressed genes among several cell types. Genes with |logFC| ≥ 0.30 and a false discovery rate (FDR or q value) < 1e−05 were considered upregulated between different cell types.

### Gene ontology and path (KEGG) enrichment analysis

Gene ontology (GO) analysis is a common method for large-scale functional enrichment studies, including CC and MF categories. The Kyoto Encyclopedia of Genes and Genomes (KEGG) is a comprehensive database for genomic, pathway, disease, and drug information. GO and KEGG enrichment analyses of ANSDR.Sig were performed using the R package clusterProfiler. The screening criteria for enriched items were adjusted p-value (adj.p) < 0.05 and FDR < 0.25, which were considered statistically significant. The Benjamini–Hochberg (BH) method was used to adjust the p-values.

### Immune-related analysis of ANSDR.Sig

To further evaluate the immunological value of ANSDR.Sig, we first calculated ANSDR.Sig scores in 30 cancer types from TCGA pan-cancer transcriptome data using the R package GSVA (version 1.44.5). Correlation with 75 published immune-related genes was analyzed based on these scores and visualized with a correlation circle diagram (12). For bulk RNA sequencing data (e.g., TCGA or ICI cohorts), normalized and, if necessary, log-transformed expression matrices (e.g., TPM or FPKM) were used as input for ssGSEA, with default parameters (method = “ssgsea”, kcdf = “Gaussian”, abs.ranking = FALSE). For single-cell RNA sequencing (scRNA-seq) data, following standard quality control and batch effect correction (using Harmony), ssGSEA was applied to the normalized expression matrix at the single-cell level.

Tumor immune microenvironment (TIME) in different cancer types was displayed according to the results of immune cell infiltration abundance. Based on the gene expression matrix, the microenvironment cell population-counter (MCPcounter) immune infiltration assay generated absolute abundance fractions of eight immune cells and two stromal cells per sample, including T cells, CD8+ T cells, cytotoxic lymphocytes, NK cells, B lymphocytes, monocytes, myeloid dendritic cells, neutrophils, endothelial cells, and fibroblasts. Based on the independent calculation results of the “MCPcounter” function in the R package IOBR, a correlation heat map was drawn for visualization.

The Molecular Signatures Database (MSigDB) was used to obtain all pathways in the HALLMARK gene set. The correlation between these pathways and ANSDR.Sig was calculated, and a correlation bubble map was drawn for visualization.

### Evaluation of clinical efficacy

The primary outcomes included objective r rate (ORR) and overall survival (OS). ORR was evaluated according to the Response Evaluation Criteria in Solid Tumors (RECIST) v1.1 in all datasets except Hugo 2016, which was evaluated using the immune-related Response Evaluation Criteria in Solid Tumors (irRECIST). Patients were divided into two groups based on response status. Complete response (CR) and partial response (PR) were defined as responders, while stable disease (SD) and progressive disease (PD) were defined as non-responders.

### Construction of immunotherapy efficacy prediction model

Five ICI RNA-seq datasets with the largest number of patient samples were combined to form a new dataset (n = 772), including RCC (n = 181), UC (n = 348), and SKCM (n = 243). The five datasets were Braun 2020 RCC, Mariathasan 2018 UC, Liu 2019 SKCM, Gide 2019 SKCM, and Riaz 2017 SKCM. The ComBat method was used to eliminate batch effects in different ICI RNA-Seq datasets. The combined dataset was randomly divided into a training set (80%, n=618) and a validation set (20%, n=154). Three additional ICI RNA-seq datasets (Zhao 2019 GBM, Snyder 2017 UC, Hugo 2016 SKCM) were used as independent validation sets (n = 68).

The immune response prediction model was trained based on ANSDR.Sig and the training set using seven common ML algorithms: naive Bayes (NB), random forest (RF), support vector machine (SVM), AdaBoost classification tree (AdaBoost), boosted logistic regression (LogitBoost), K-nearest neighbors (KNN), and the cancerclass algorithm. For each ML algorithm except cancerclass, five-fold cross-validation was used for hyperparameter tuning to optimize model performance. To ensure robustness, the optimization process was repeated 10 times using different random seeds. For the cancerclass algorithm, the entire training set was used to train the model.

The ML algorithms were used to construct prediction models based on the training set. The area under the curve (AUC) was calculated using the validation set, and the best-performing ML algorithm was selected. The final prediction model of immunotherapy efficacy related to the autonomic nervous system was then constructed. When AUC > 0.5, it indicated a trend toward promoting event occurrence. The closer the AUC value was to 1, the better the diagnostic effect. AUC between 0.5 and 0.7 indicated low accuracy, between 0.7 and 0.9 moderate accuracy, and above 0.9 high accuracy.

To further compare the predictive performance of ANSDR.Sig, we compared its AUC values with those of six previously reported ICI response–related gene sets (INFG.Sig, T.cell.inflamed.Sig, PDL1.Sig, LRRC15.CAF.Sig, NLRP3.Sig, Cytotoxic.Sig) in the training set, validation set, and three independent validation sets.

### CRISPR profiling

To explore potential therapeutic targets for ANSDR.Sig, we collected data from seven published CRISPR/Cas9 screening studies that assessed the individual effects of gene knockdown on tumor immunity, including Freeman 2019 (13), Kearney 2018 (14), Manguso 2017 (15), Pan 2018 (16), Patel 2017 (17), Vredevoogd 2019 (18), and Lawson 2020 (19). Data from these studies were divided into 17 datasets based on the model cell lines and treatment conditions applied. The CRISPR analysis involved SKCM, GBM, CRC, and RCC cell lines. We used these data to identify genes in different datasets that are more likely to regulate lymphocytes and influence response to immunotherapy.

CRISPR screening was performed by genome wide CRISPR–Cas9 knockout in different cancer cell lines. Cell lines from different cancers were cultured with or without cytotoxic lymphocytes (CTLs) *in vitro* and also implanted into immunodeficient or immunocompetent mice *in vivo*. The abundance of sgRNAs for the corresponding genes was evaluated by RNA sequencing. To measure the impact of gene knockout on cancer under CTL or anti-tumor immunity, logFC values were calculated between different cell line groups. To eliminate batch effects and enable cross-study comparisons of gene expression in different CRISPR datasets, we adopted a z score normalization method based on logFC values. First, for each gene, we calculated its logFC between experimental groups within each CRISPR dataset to capture expression changes under knockout versus control conditions. Subsequently, z scores were computed for each gene across datasets using the formula $\text{z }-\text{secore }=\;\frac{X-\mu }{\sigma }$ . X represents the logFC value of the gene in a specific dataset, μ is the mean logFC of that gene across all samples or datasets, and σ is the corresponding standard deviation. This approach allowed for more accurate comparisons of gene expression changes across distinct CRISPR datasets, effectively mitigating the influence of inter-sample variability. As a result, gene expression alterations from different studies could be systematically integrated and analyzed. Lower z scores indicated better immune response after gene knockdown. We ranked genes based on the mean z scores across the 17 datasets; the top -ranked genes with lower z scores were considered immune resistance genes.

To evaluate the predictive value of ANSDR.Sig, we compared it with previously reported ICI response–associated genes, which consisted of one pancancer–related signature (LRRC15.CAF.Sig) and four SKCM -specific signatures (CRMA.Sig, ImmuCells.Sig, IMS.Sig, and TRS.Sig). Based on the immune response prediction results in the training set, the correlation between ANSDR.Sig and the pan-cancer –related signature gene list was analyzed. The relationship between ANSDR.Sig and SKCM-specific signature gene lists was assessed based on the results of immunotherapy efficacy prediction in independent training sets (Hugo 2016 and Van Allen 2015).

### Machine learning algorithm to screen out Hub-ANSDR.Sig

In order to explore the relationship between ANSDR.Sig and prognosis, we constructed a prognostic risk model using five ML algorithms: bagged trees, Bayesian, LQV, Boruta, and random forest (RF). The final set of genes was obtained by intersecting the results of the five algorithms. Genes that appeared in at least three algorithms were defined as Hub-ANSDR.Sig.

The R package survival was used to perform Kaplan–Meier (KM) survival analysis to evaluate differences in overall survival (OS) between high- and low -risk groups. KM curves for the training and validation sets were generated based on RiskScore.

### Panoramic analysis of Hub-ANSDR.Sig

The R package GSVA was used to calculate the autonomic nervous system–related score based on the expression matrix of Hub-ANSDR.Sig and the TCGA pan-cancer transcriptome dataset using the ssGSEA algorithm. This was done to evaluate the correlation between Hub-ANSDR.Sig and immune infiltration in various cancer types. We then analyzed the correlation between the autonomic nervous system –related score, microsatellite instability (MSI), and immune checkpoint expression.

To explore the impact of Hub-ANSDR.Sig on anti-tumor capacity in pan-cancer, we analyzed the correlation between OS and Hub-ANSDR.Sig across different cancer types and plotted corresponding KM curves. Based on these results, we identified a representative cancer type for in-depth analysis.

### Construction and correlation analysis of STAD subtypes

Consensus clustering is a resampling-based algorithm to identify each member and its subgroup number for verification of the rationality of clustering. Consensus clustering is a series of iterations on subsamples of the dataset. It applies subsampling to induce sampling variability in order to provide an indicator of cluster stability and parameter decision. The consensus clustering method of the R package ConsensusClusterPlus was used to identify different subtypes of STAD based on Hub-ANSDR.Sig. In this process, the number of clusters was set between two and six, and 80% of the total samples were sampled with 1,000 repetitions, clusterAlg = KM, distance = Euclidean.

To obtain the dysfunction scores of different subtypes of STAD, the analysis was performed by the tumor immune dysfunction and exclusion (TIDE) algorithm in the expression matrix of STAD samples. Next, we calculated the group difference of dysfunction score, MSI, and TMB scores in different subtypes of STAD by Mann–Whitney U Test (Wilcoxon Rank Sum Test).

### Somatic mutation analysis of STAD subtypes

The “Masked Somatic Mutation” data were selected as the somatic mutation data of STAD samples through the TCGA website, and the data were preprocessed by VarScan software. Finally, the R package maftools were used to visualize the somatic mutation in different subtypes of STAD.

### Immune infiltration analysis of STAD subtypes

CIBERSORT is based on linear support vector regression to deconvolute the transcriptome expression matrix, aiming to estimate the composition and abundance of immune cells in a mixture of cells. The CIBERSORT algorithm combined with the LM22 feature gene matrix was used to filter out the data with an immune cell enrichment score greater than zero. The specific results of the immune cell infiltration matrix were finally obtained. The R package ggplot2 was used to draw group comparison maps to show the expression differences of immune cells in different subtypes of STAD. The R package pheatmap was used to draw heat maps to show the correlation between immune cells and Hub-ANSDR.Sig in different STAD subtypes. Based on the correlation between Hub-ANSDR.Sig and immune cells, the results with p -value < 0.05 were screened, and the correlation dot plots with the top two positive and negative correlations were drawn.

Single-sample gene-set enrichment analysis (ssGSEA), known as single-sample gene-set enrichment analysis, quantifies the relative abundance of each immune cell infiltration. Firstly, each infiltrating immune cell type was labeled. Secondly, the enrichment scores calculated using ssGSEA analysis were used to represent the relative abundance of each immune cell infiltration in each sample, and the samples with p -value < 0.05 were filtered out to obtain the immune cell infiltration matrix. Then, the R package ggplot2 was used to draw group comparison maps to show the expression differences of immune cells in different subtypes of STAD. Finally, the correlation between Hub-ANSDR.Sig and immune cells was calculated, and the results with p -value < 0.05 were filtered out. The R package heatmap was used to draw the correlation dot plot for display.

### Construction of regulatory network

Transcription factors (TFs) control gene expression at the post-transcriptional stage by interacting with Hub-ANSDR.Sig. The TFs in the ChIPBase database were searched to analyze the regulatory effects of TFs on Hub-ANSDR.Sig. Cytoscape software was used to visualize the mRNA–TF regulatory network. In order to analyze the relationship between Hub-ANSDR.Sig and microRNAs, the miRNAs linked with Hub-ANSDR.Sig were obtained from the TarBase database. The mRNA–miRNA regulatory network was visualized by Cytoscape software.

### Cell communication analysis

Cell–cell communication was inferred and quantified by combining single-cell expression profiles with known ligands, receptors, and their cofactors from the CellPhoneDB.human ligand –receptor database using the R package CellChat. Significant interaction pairs were further identified based on ligand–receptor interaction probability and perturbation tests. The cell–cell communication network was then integrated by counting the number or strength of significant ligand–receptor relationship pairs between cell types. A heat map and a circle chart were used to display the number and strength of interactions, respectively. A bubble plot was used to show all important receptor–ligand pairs involved in immune cell signaling.

### Pseudotime analysis

According to the temporal gene expression of each cell, cells can be arranged along a trajectory corresponding to pseudotime (20). Based on gene expression patterns, samples can be divided into multiple cell populations in distinct differentiation states, and a lineage development dendrogram can be generated to predict the developmental and differentiation trajectories of cells. The pseudotime results require confirmation of the origin and endpoint of differentiation by assessing the distribution of cell types along the trajectory and the expression dynamics of signature genes. In this study, we used pseudotemporal analysis to predict the developmental trajectory of GSE134520 and to analyze the variation of Hub-ANSDR.Sig over the pseudotemporal course.

### Clinical specimen acquisition

Paired gastric cancer and para-cancer specimens were obtained from fresh clinical tissues, quickly washed with 0.9% sodium chloride solution, and frozen in liquid nitrogen within 5 m. Clinical information is shown in **Supplementary Table S5**. Paraffin sections of tissues with different immunotherapy efficacy (clinical information presented in **Supplementary Table S6**) were obtained from the Department of Pathology, Jiangsu Province Hospital of Chinese Medicine.

### RT-PCR assay

Total RNA was extracted from clinical tissue samples using RNA Extraction Reagent (Servicebio, Wuhan, China) and reverse-transcribed into cDNA using SweScript All-in-One SuperMix with gDNA Remover (Servicebio, Wuhan, China). The primers used are listed in **Supplementary Table S4**. Gene expression was analyzed using a real-time PCR system (Bio-Rad, California, USA) with Universal Blue SYBR Green qPCR Master Mix (Servicebio, Wuhan, China). The △△Ct method was used for result analysis.

### Immunohistochemical staining

Paraffin sections were incubated in xylene and dehydrated in pure ethanol, followed by dehydration in gradient ethanol. After that, all sections were washed in distilled water. Slides were immersed in sodium citrate antigen retrieval solution (pH 6.0). After natural cooling, sections were washed with PBS (pH 7.4) using a rocker device. Sections were then immersed in 3% H_2_O_2_ and incubated at room temperature away from light, followed by another PBS wash (pH 7.4). Objective tissues were covered with 3% BSA at room temperature for 30 min. Slides were incubated with primary antibody overnight at 4°C. After washing with PBS, objective tissues were covered with secondary antibody labeled with HRP and incubated at room temperature for 50 min. DAB chromogenic reagent was added, and the reaction time was monitored under a microscope until the nucleus appeared brown-yellow. Counterstaining of the nucleus was performed using hematoxylin staining solution. Slides were then dehydrated successively in gradient ethanol, pure ethanol, and xylene, followed by mounting with resin mounting medium. For result interpretation, nuclei appeared blue, and positive cells were characterized by brown-yellow staining. IHC images were analyzed using ImageJ. Images were converted to 8-bit grayscale, and optical density was calibrated via Uncalibrated OD. Thresholds were adjusted to select DAB-positive areas. Mean gray values were measured and recorded as average optical density (AOD). Data were exported for statistical analysis of protein expression levels.

### Statistical analysis

Data processing and bioinformatics analyses were performed using R software (version 4.2.0). Continuous variables are presented as mean ± standard deviation (SD). The Wilcoxon rank-sum test was used for comparisons between two groups. Unless otherwise specified, correlation coefficients between different molecules were calculated using Spearman’s correlation analysis. Additional statistical analyses were conducted using IBM SPSS Statistics for Windows, version 23.0 (IBM Corp., Armonk, NY, USA), with one-way ANOVA followed by Duncan’s test. A p-value < 0.05 was considered statistically significant.

## Results

### Association between ANSD-related genes and tumor immunotherapy efficacy

**Figure 2:**
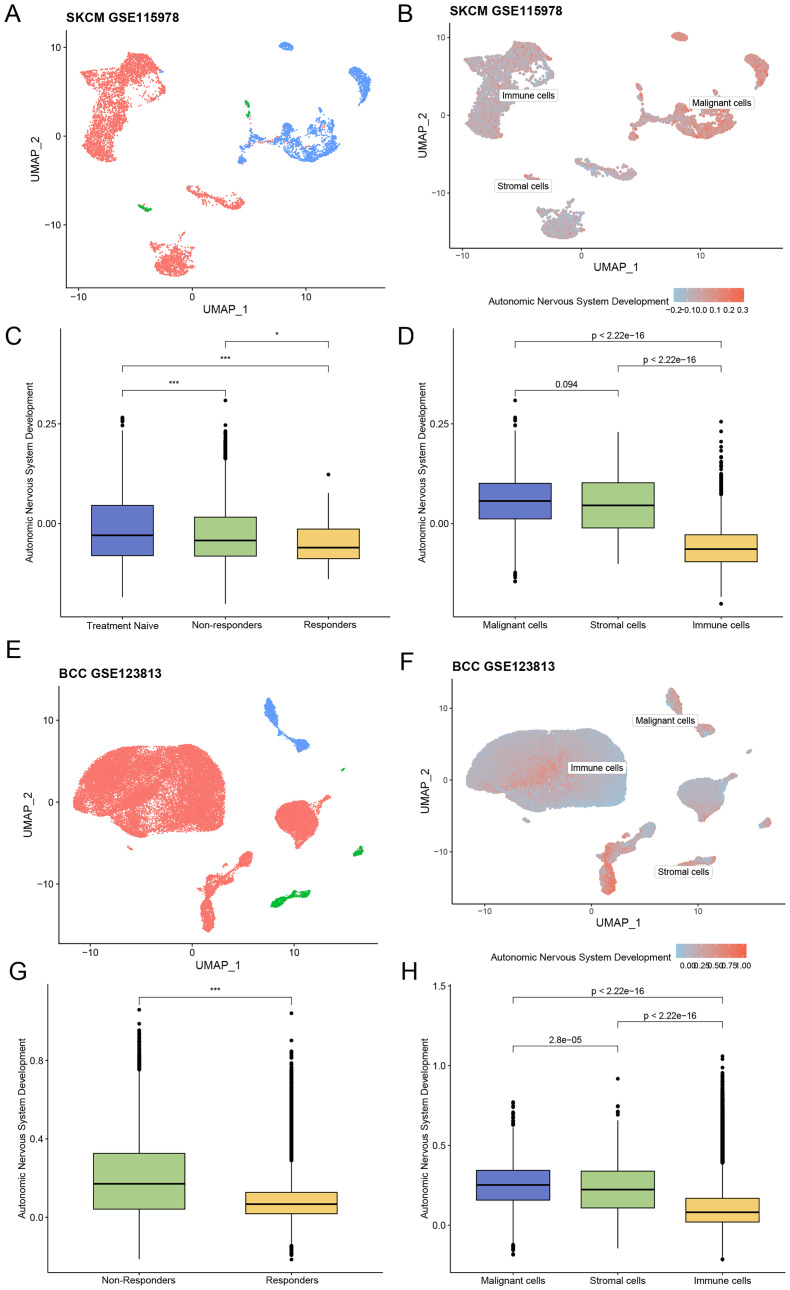
Association between ANSD-related genes and ICI outcomes. **(A)** Distribution of different cell subtypes in the SKCM dataset GSE115978. **(B)** Enrichment score of ANSD -related genes in GSE115978. **(C)** Relationship between immunotherapy efficacy and ANSD score in GSE115978. **(D)** Differences in ANSD scores among different cell subtypes in GSE115978. **(E)** Distribution of different cell subtypes in the BCC dataset GSE123813. **(F)** Enrichment score of ANSD -related genes in GSE123813. **(G)** Relationship between immunotherapy efficacy and ANSD score in GSE123813. **(H)** Differences in ANSD scores among different cell subtypes in GSE123813. *p<0.05, ***p<0.001.

The skin cutaneous melanoma (SKCM) dataset GSE115978 included 32 patients after quality control: 15 in the treatment -naive (TN) group, 15 in the immunotherapy non-responder (NR) group, and one in the responder (R) group. The distribution of immune, stromal, and malignant cells and the corresponding ANSD scores in GSE115978 were visualized using UMAP (**Figures 2A, B**). Group comparisons showed the diversity of ANSD scores across different immunotherapy efficacy groups and cell subtypes. The results revealed that the ANSD score in group R was lower than in group NR (p<0.05) and group TN (p<0.001). Compared with group TN, group NR also showed a lower ANSD score (p<0.001). Additionally, ANSD-related genes were more highly enriched in malignant cells than in immune or stromal cells (**Figures 2C, D**).

A total of 10 patients were included in the basal cell carcinoma (BCC) dataset GSE123813 after quality control, consisting of four in group NR and six in group R. The distribution of cell types and corresponding ANSD scores were presented by UMAP (**Figures 2E, F**). As shown, the ANSD score in group R was significantly lower than in group NR (p<0.001), and ANSD -related genes were again highly enriched in malignant cells (**Figures 2G, H**).

### Screening of ANSDR.Sig and immune correlation analysis

**Figure 3:**
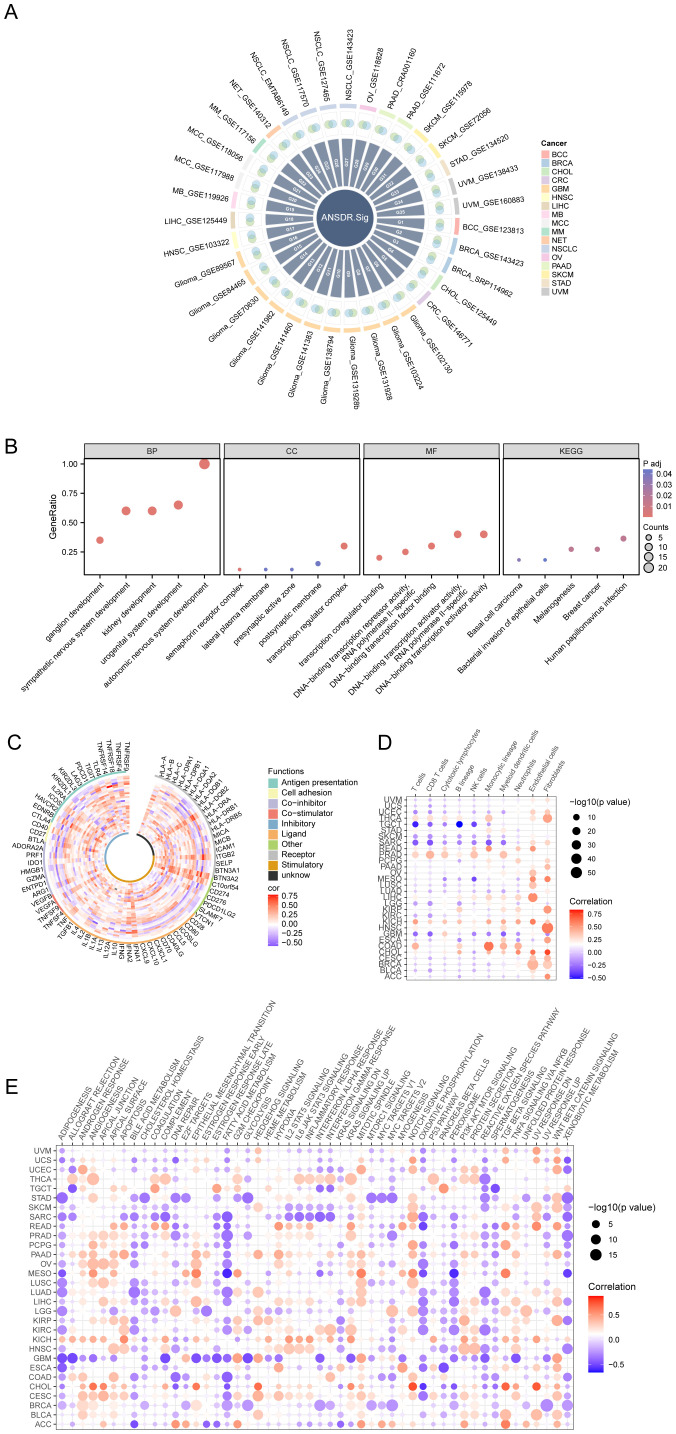
Development and immune analysis of ANSDR.Sig. **(A)** Intersection circle diagram of genes positively related to ANSD score and upregulated genes in malignant tumors in the scRNA-seq dataset. **(B)** Bubble plot of GO and KEGG enrichment analysis results of ANSDR.Sig. The screening criteria were adj.p<0.05 and FDR (q value)<0.25. **(C)** Correlation circle diagram of ANSDR.Sig and immune-related genes. **(D)** Dot plot of correlation between ANSDR.Sig and immune infiltration. **(E)** Dot plot of correlation between ANSDR.Sig and HALLMARK -associated pathway.

Thirty-four scRNA-seq datasets were selected to screen ANSD -related genes with differential expression. The relationship between ANSD -related genes and ANSD scores in malignant cells across the 34 scRNA-seq datasets was calculated by Spearman correlation analysis. Genes positively correlated with ANSD score (Spearman r > 0.3 and p < 0.05) were labeled as Gx, and genes with higher expression in malignant cells were recorded as Gy. To obtain upregulated, cancer-specific genes positively associated with ANSD score, Gx and Gy were intersected to generate Gn. Genes obtained from Gn in each dataset were merged and deduplicated. ANSDR.Sig, consisting of 20 genes—CTNNB1, GATA3, NRP2, SOX4, TFAP2A, TP63, HES1, SOX11, ASCL1, FZD3, NAV2, SOX8, FN1, INSM1, TFAP2B, NF1, NRP1, SIX1, EDNRB, and SOX10—was ultimately acquired (**Figure 3A**).

GO and KEGG enrichment analyses were conducted to assess the biological processes (BP), molecular functions (MF), cellular components (CC), and biological pathways associated with ANSDR.Sig (**Supplementary Table S1**). The results showed that ANSDR.Sig is mainly enriched in BP categories including autonomic nervous system development, sympathetic nervous system development, urogenital system development, ganglion development, and kidney development. The related CC terms included transcription regulator complex, semaphorin receptor complex, lateral plasma membrane, postsynaptic membrane, and presynaptic active zone. The corresponding MF terms covered DNA-binding transcription activator activity, transcription coregulator binding, DNA-binding transcription factor binding, and DNA-binding transcription repressor activity. ANSDR.Sig was also enriched in multiple biological pathways such as melanogenesis, human papillomavirus infection, breast cancer, basal cell carcinoma, and bacterial invasion of epithelial cells. The results were visualized using bubble plots (**Figure 3B**).

We further explored the correlation between ANSDR.Sig and 75 immune-related genes based on ANSD scores in 30 cancer types from the TCGA pan-cancer transcriptome dataset. The results showed that ANSDR.Sig is positively correlated with the majority of immune-related genes (**Figure 3C**). Evaluation of immune cell infiltration in pan-cancer demonstrated that ANSDR.Sig generally contributes positively to immune cell infiltration. Notably, ANSDR.Sig was positively correlated with immune cell infiltration in head and neck squamous cell carcinoma (HNSC) and kidney chromophobe carcinoma (KICH), while showing negative correlations in testicular germ cell tumors (TGCT) and sarcoma (SARC) (**Figure 3D**). Subsequent analysis of the relationship between HALLMARK pathways and ANSDR.Sig in the TCGA pan-cancer dataset revealed that ANSDR.Sig is negatively correlated with several biological pathways associated with immunosuppression, including ADIPOGENESIS, BILE ACID METABOLISM, FATTY ACID METABOLISM, OXIDATIVE PHOSPHORYLATION, SPERMATOGENESIS and XENOBIOTIC METABOLISM (**Figure 3E**).

### Construction of immunotherapy efficacy prediction model and CRISPR analysis

**Figure 4:**
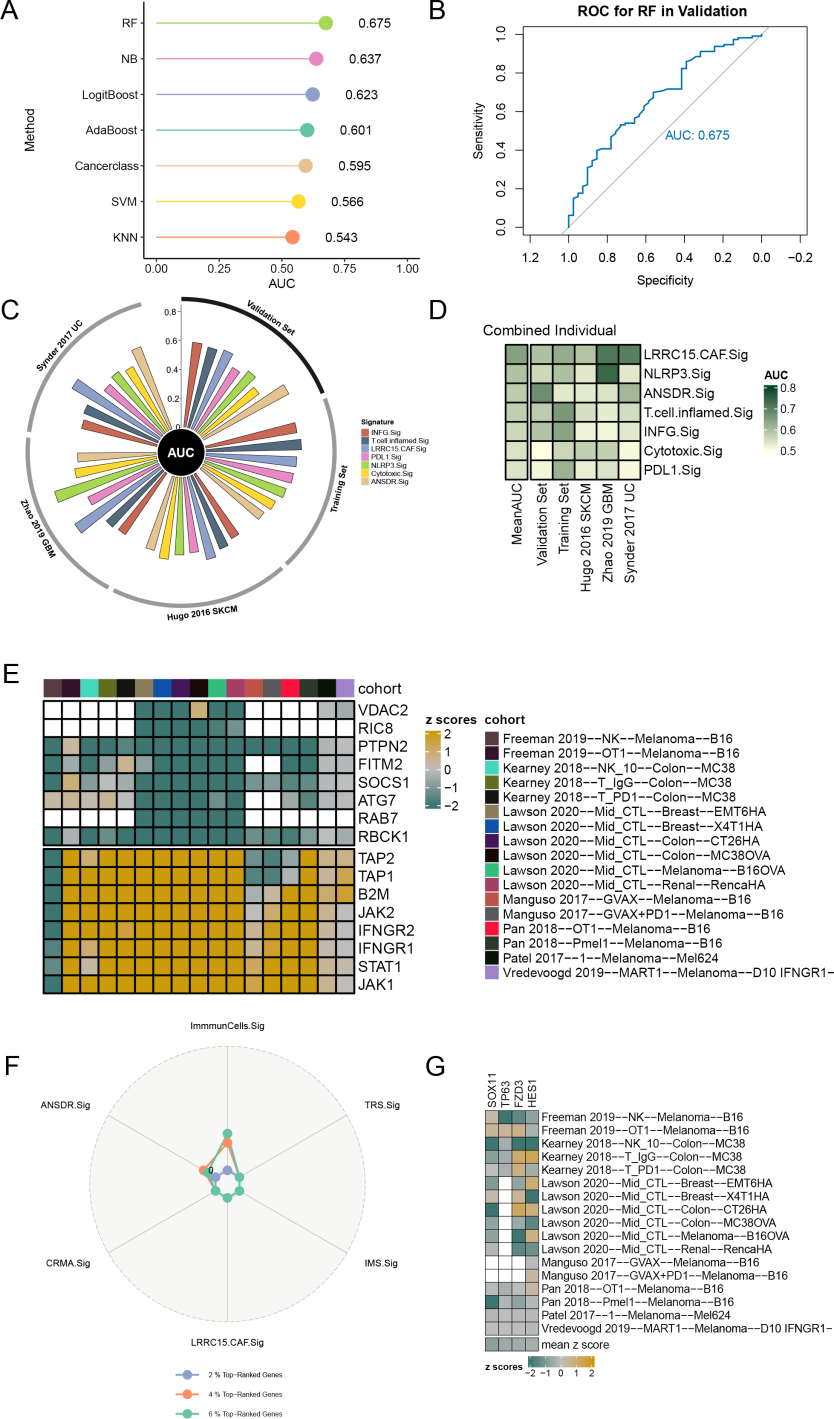
Prediction of ICI outcomes and CRISPR analysis of ANSDR.Sig. **(A)** Performance of seven ML algorithms used to construct prediction models for immunotherapy efficacy. **(B)** ROC curve of the prediction model constructed by the RF algorithm. **(C)** Circle plot showing the performance of different ICI RNA-seq datasets in predicting immune response. **(D)** Heat map of the ability of ICI response signature–associated genes to predict immune response. **(E)** Genes ranked based on 17 CRISPR datasets and z scores. Green indicates immune resistance genes; yellow represents immune-sensitive genes; white indicates missing gene data from the core dataset. **(F)** Radar plot of the percentage of top-ranked genes in ANSDR.Sig and ICI response signature–associated gene sets. **(G)** Heat map visualization of the z scores for four genes in ANSDR.Sig.

In order to investigate the relationship between ANSDR.Sig and immunotherapy efficacy, we collected eight ICI RNA-seq datasets with clear immunotherapy efficacy and clinical information. The training set was used to construct immune response prediction models via seven ML algorithms. The best prediction model was selected by comparing the AUC values, and 5-fold cross -validation with 10 replications was performed on each model for parameter optimization. The results showed that the prediction model constructed by the random forest (RF) algorithm had greater predictive ability (AUC = 0.675) (**Figures 4A, B**). Next, to validate the potential of ANSDR.Sig in predicting immunotherapy efficacy, ANSDR.Sig was compared with six ICI response characteristic gene sets in three published RNA-seq datasets. The results showed that the predictive ability of ANSDR.Sig was better in the validation sets and in Synder 2017UC (**Figures 4C, D**).

Data from seven CRISPR studies were systematically collected and further divided into 17 CRISPR datasets, with a total of 22,505 genes recorded. Genes were first ranked according to their average z scores. The top -ranked genes were immune resistance genes, speculated to promote anti-tumor immunity upon knockdown. The lowest -ranked genes were immune-sensitive genes, which may inhibit anti-tumor immunity when knocked out (**Figure 4E**). Among them, the numbers of genes in the top 2%, 4%, and 6% were 450, 900, and 1,350, respectively. The percentage of top-ranked genes in ANSDR.Sig and ICI response signature-associated gene sets was then calculated. The results showed that the percentage in ANSDR.Sig was higher than that in most ICI response signature-associated gene sets, except for the SKCM -specific signature ImmuCells.Sig (**Figure 4F**). The top 20% of ranked genes included four ANSDR.Sig members—SOX11, TP63, FZD3, and HES1. These four genes were identified as immune resistance genes and validated across 17 CRISPR datasets, suggesting their potential as predictive targets for immunotherapy (**Figure 4G**).

### Screening of Hub-ANSDR.Sig and KM curve analysis

**Figure 5:**
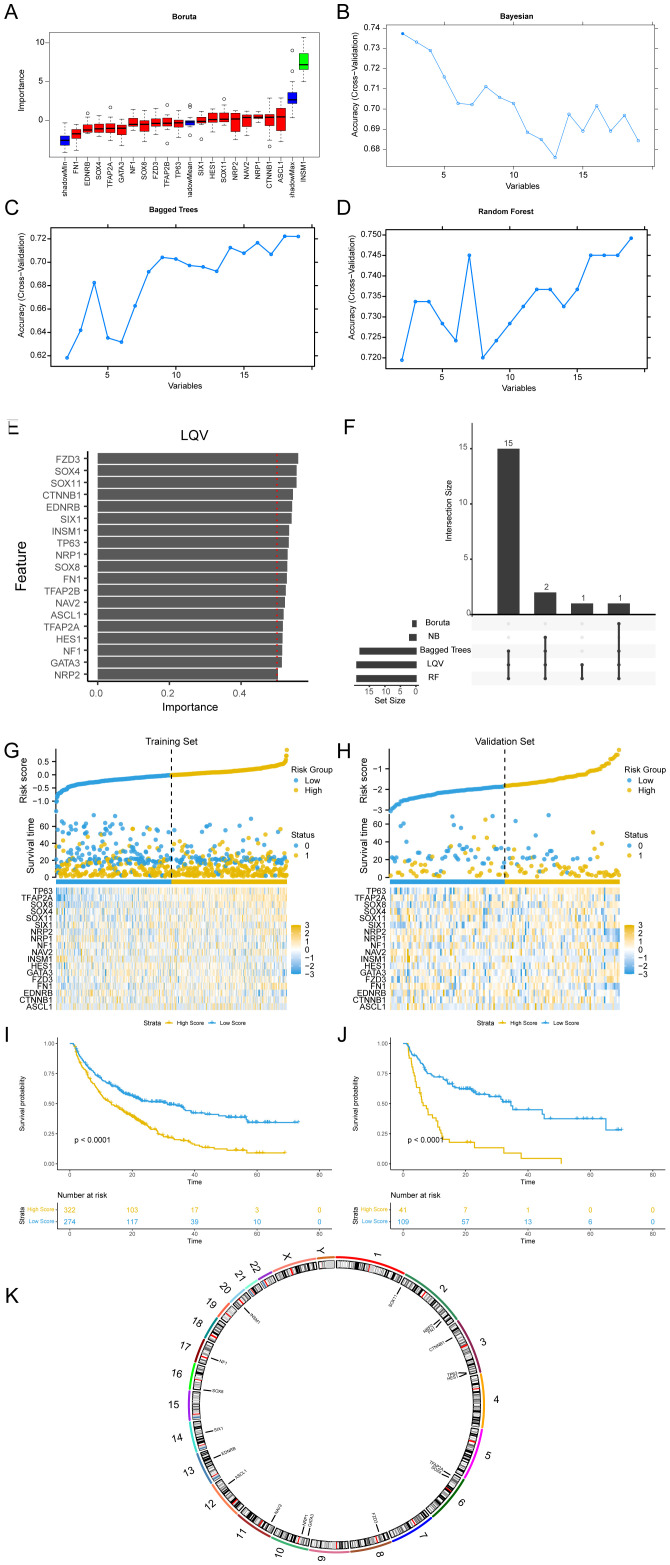
Machine learning and Hub-ANSDR.Sig analysis. **(A)** Boruta algorithm prognostic risk model of ANSDR.Sig. **(B)** Bayesian algorithm prognostic risk model map of ANSDR.Sig. **(C)** Bagged Trees algorithm prognostic risk model diagram of ANSDR.Sig. **(D)** RF algorithm prognostic risk model diagram of ANSDR.Sig. **(E)** LQV prognostic risk model plot of ANSDR.Sig. **(F)** Intersection plot of results from five ML algorithms. **(G)** Risk factor plots of Hub-ANSDR.Sig and outcomes in the training set. **(H)** Prognostic KM curve between the RiskScore and OS of training set samples. **(I)** Map of Hub-ANSDR.Sig and risk factors for prognosis in the validation set. **(J)** Prognostic KM curve between the RiskScore and OS of validation set samples. **(K)** Chromosomal mapping of Hub-ANSDR.Sig.

Five ML algorithms, including the wrapper (Boruta) algorithm (**Figure 5A**), Bayesian algorithm (**Figure 5B**), bagged trees algorithm (**Figure 5C**), RF algorithm (**Figure 5D**), and learning vector quantization (LQV) algorithm (**Figure 5E**), were applied to analyze the relationship between ANSDR.Sig and immunotherapy efficacy based on the training set. The results from the different ML algorithms were intersected (**Figure 5F**). As shown, genes that appeared in at least three ML algorithms were defined as Hub-ANSDR.Sig, and a total of 18 genes were acquired: ASCL1, CTNNB1, EDNRB, FN1, FZD3, GATA3, HES1, INSM1, NAV2, NF1, NRP1, NRP2, SIX1, SOX11, SOX4, SOX8, TFAP2A, and TP63.

Using multivariate Cox regression coefficients, the RiskScore of all samples in the training and validation sets was calculated based on Hub-ANSDR.Sig. Samples in both datasets were divided into high- and low -score groups using the optimal cut-off value determined by the “surv_categorize” function. The relationship between different samples was displayed using a risk factor graph. Kaplan–Meier (KM) curves for the training and validation sets were plotted based on RiskScore. The results showed that Hub-ANSDR.Sig was associated with overall survival (OS) in both training and validation cohorts (**Figures 5G–J**). The R package RCircos was used to analyze the chromosomal location of Hub-ANSDR.Sig to generate a chromosome localization map. The mapping results showed that most Hub-ANSDR.Sig genes—including SOX11, NRP2, FN1, CTNNB1, TP63, and HES1—are located on chromosomes 2 and 3 (**Figure 5K**).

### Panoramic analysis of Hub-ANSDR.Sig

**Figure 6:**
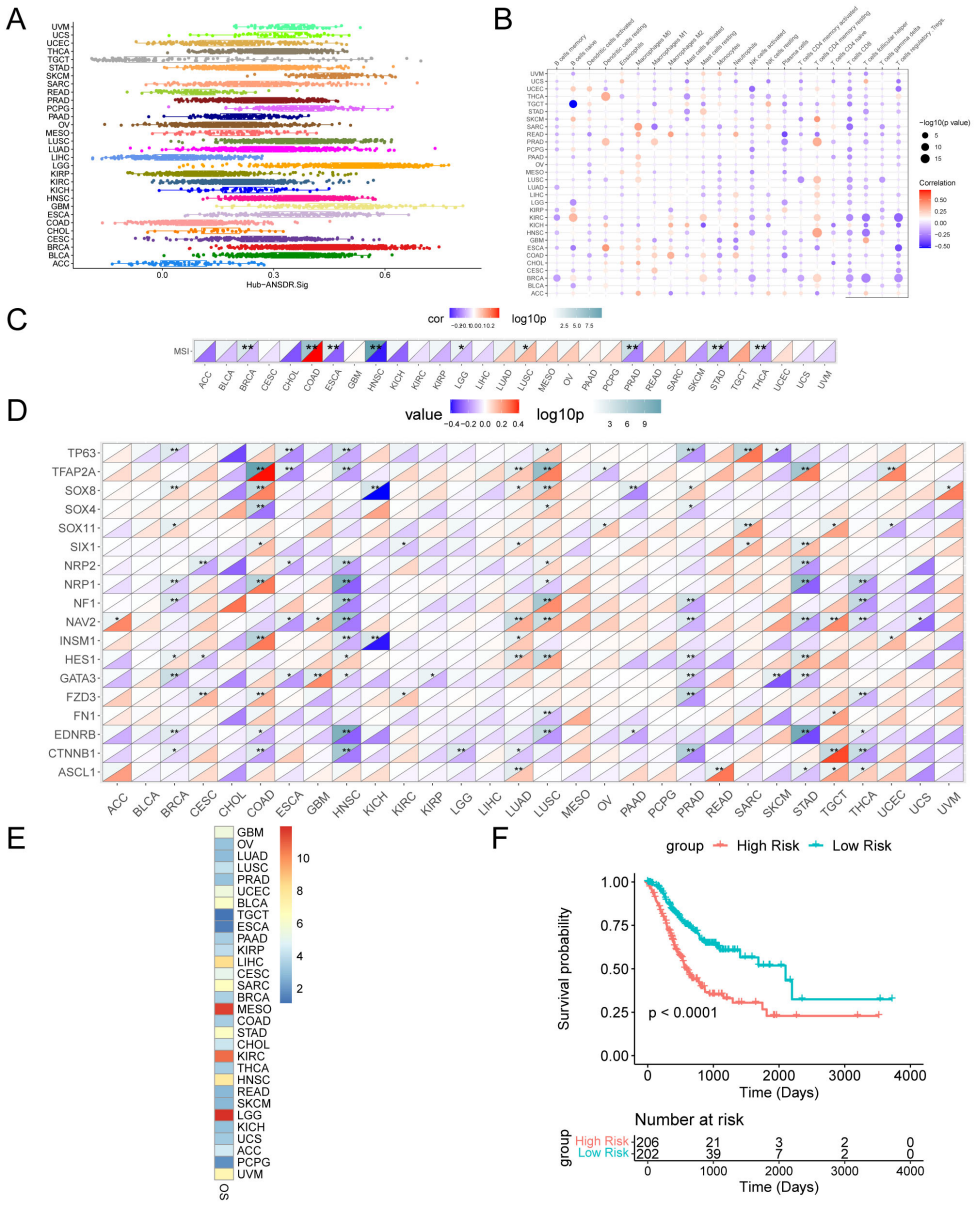
Landscape analysis of Hub-ANSDR.Sig. **(A)** Hub-ANSDR.Sig scores in different cancer types. **(B)** Correlation between Hub-ANSDR.Sig and immune cell infiltration abundance. **(C)** Correlation between Hub-ANSDR.Sig and MSI. **(D)** Correlation between genes from Hub-ANSDR.Sig and MSI. **(E)** Association between Hub-ANSDR.Sig and OS. **(F)** KM curve between the risk score of Hub-ANSDR.Sig and OS in STAD. *p<0.05, **p<0.01.

The scores of Hub-ANSDR.Sig in 30 cancer types from the TCGA dataset were evaluated by single-sample gene-set enrichment analysis (ssGSEA) (**Figure 6A**). The correlation between Hub-ANSDR.Sig and immune cell infiltration abundance was then analyzed across different cancer types. The results showed that Hub-ANSDR.Sig is positively correlated with resting memory CD4^+^ T cells but negatively linked with CD8^+^ T cells and regulatory T cells (Tregs) in most cancer types (**Figure 6B**). Further analysis of the correlation between Hub-ANSDR.Sig and MSI in 30 different cancers revealed that Hub-ANSDR.Sig has the strongest positive association with MSI in colon adenocarcinoma (COAD), while the most negative correlation was observed in HNSC (**Figure 6C**). The role of each gene from Hub-ANSDR.Sig in MSI was also examined across 30 cancer types. TFAP2A showed the strongest positive association with MSI in COAD, whereas SOX8 presented the strongest negative correlation in KICH (**Figure 6D**).

A KM curve was conducted to analyze the effect of Hub-ANSDR.Sig on cancer prognosis. The results showed that Hub-ANSDR.Sig is significantly associated with OS in patients with brain lower -grade glioma (LGG), mesothelioma (MESO), and stomach adenocarcinoma (STAD) (**Figure 6E**). The association between the Hub-ANSDR.Sig –derived risk score and OS in STAD patients was then explored. The KM curve showed a significant difference in OS between high- and low -risk score groups (**Figure 6F**).

### Regulatory network, cell communication, and pseudotime analysis of Hub-ANSDR.Sig

**Figure 7:**
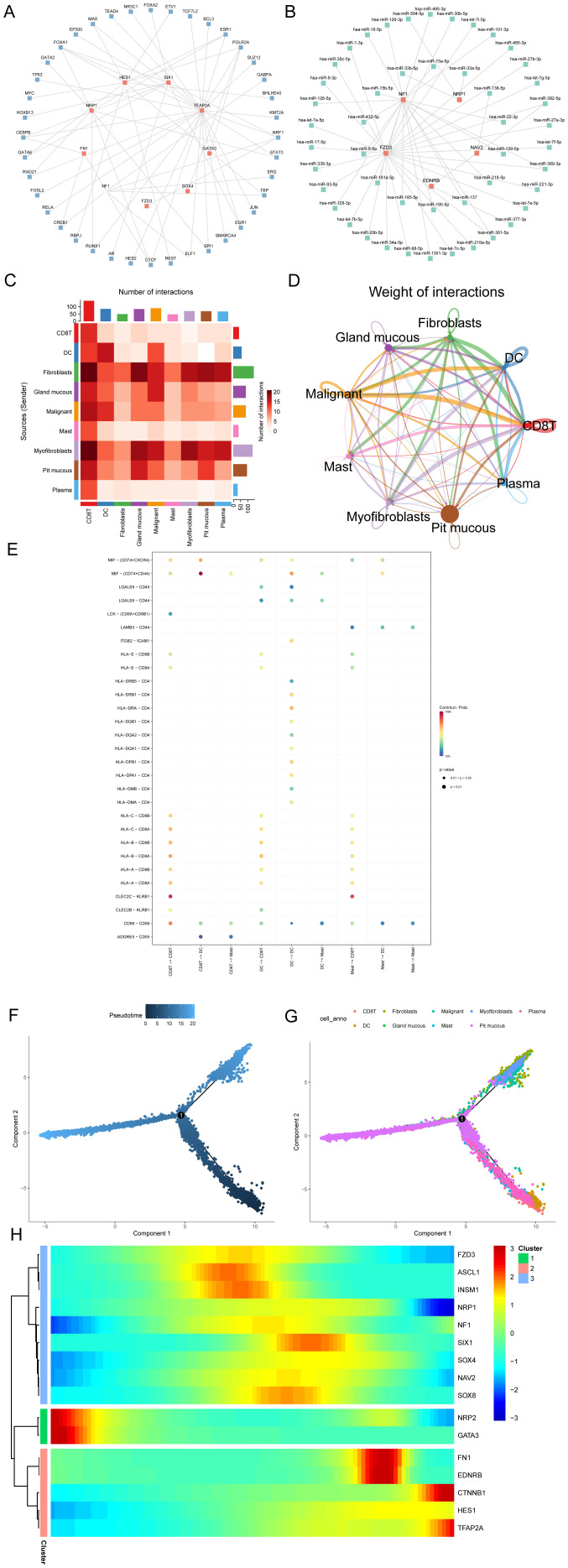
Regulatory network, cell communication, and pseudotime analysis. **(A)** mRNA-TF regulatory network of Hub-ANSDR.Sig. **(B)** mRNA–miRNA regulatory network of Hub-ANSDR.Sig. **(C)** Heatmap showing the number of interaction relationship pairs between different cell types. **(D)** Network diagram showing interaction strength between different cell types. **(E)** Receptor–ligand pairs involved in cell communication among immune cell populations. **(F)** Time-sequence diagram of the differentiation and development of different cell types. **(G)** Plot of the differentiation and development trajectories of different cell types. **(H)** Pseudotime classification heat map of Hub-ANSDR.Sig.

Transcription factors (TFs) that bind to Hub-ANSDR.Sig were obtained from the ChIPBase database. The mRNA–TF regulatory network was then constructed and visualized (**Figure 7A**). Notably, nine important genes in Hub-ANSDR.Sig and 40 TFs were involved, with detailed information provided in **Supplementary Table S2**. MicroRNAs related to Hub-ANSDR.Sig were retrieved from the TarBase database. The mRNA–miRNA regulatory network was subsequently established and visualized (**Figure 7B**), involving five Hub-ANSDR.Sig molecules and 48 miRNAs, which are listed in **Supplementary Table S3**.

The STAD scRNA-seq dataset GSE134520 and related cell annotation information were collected from the TISCH database. The R package CellChat was used to infer and quantify communication between different cell types. A heat map and a circle graph were used to visualize the number and intensity of cell–cell communications (**Figures 7C, D**). The results showed that the number of interactions between cytotoxic T cells (CD8T) and fibroblasts was notably higher. Among three immune cell types (CD8T, dendritic cells [DC], and mast cells), key receptor–ligand pairs involved in sending or receiving signals were identified. The MIF–(CD74+CD44) receptor–ligand pair showed the strongest interaction strength between CD8T and dendritic cells (DC) (**Figure 7E**).

Pseudotime analysis was performed to explore the developmental trajectories of different cell types, which were visualized using differentiation sequence and trajectory plots (**Figures 7F, G**). Based on these results, we inferred that cytotoxic T cells, mast cells, and dendritic cells are positioned at the early stages of the developmental trajectory, followed by gastric epithelial cells (pit mucous), myofibroblasts, and fibroblasts. To identify genes with similar kinetic trends, Hub-ANSDR.Sig genes were classified, and the expression patterns of three gene groups over pseudotime were visualized using a heat map (**Figure 7H**). Each row represented one gene, and each column represented the average expression value in the current cell state. Expression values were color-coded from red to blue to indicate decreasing expression.

### Construction and correlation analysis of gastric cancer subtypes

**Figure 8:**
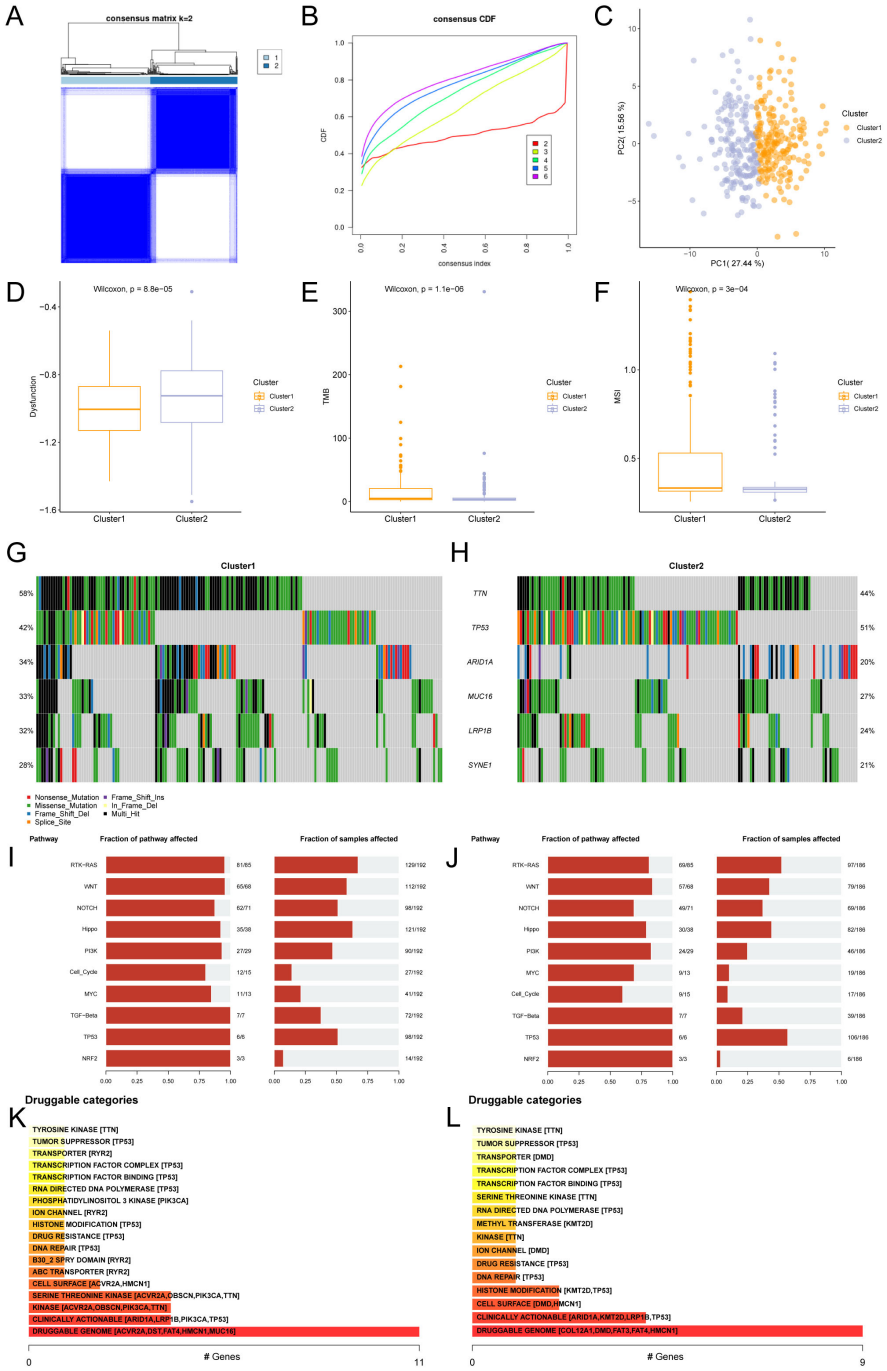
Consensus cluster and related analysis of STAD subtypes. **(A)** Consensus cluster analysis results of STAD samples. **(B)** Consistency cumulative distribution function (CDF) plot of consensus clustering. **(C)** PCA plot of the two subtypes of STAD. **(D–F)**. Group comparison of dysfunction **(D)**, TMB **(E)**, and MSI **(F)** scores between STAD subtypes. **(G, H)** Mutational gene landscape based on Hub-ANSDR.Sig in STAD. **(I)** Analysis of biological functions affected by mutations in subtype A (Cluster1). **(J)** Analysis of biological functions affected by mutations in subtype B (Cluster2). **(K)** Classification of potentially druggable genes in subtype A (Cluster1). **(L)** Classification of potentially druggable genes in subtype B (Cluster2). Each category is followed by the top five genes in parentheses. The y-axis represents druggable genes in each category.

The R package ConsensusClusterPlus was used to identify different STAD subtypes based on the expression of Hub-ANSDR.Sig in the TCGA-STAD dataset through consensus clustering analysis. Two subtypes of STAD were ultimately determined and defined as Cluster1 and Cluster2 (**Figures 8A, B**). Cluster1 (subtype A) contained 196 samples, and Cluster2 (subtype B) contained 192 samples. The PCA plot showed a clear separation between the two subtypes, indicating significant differences (**Figure 8C**). In addition, dysfunction, TMB, and MSI scores differed significantly between the two STAD subtypes (**Figures 8D–F**).

Gene mutation rates were analyzed using the R package maftools based on genes with the highest mutation frequency in Cluster1 and Cluster2. The results showed that TTN had the highest mutation frequency (58%) in subtype A (Cluster1), while TP53 had the highest mutation frequency (51%) in subtype B (Cluster2) (**Figures 8G, H**). The biological function variations caused by these mutations were then examined. Subtype A (Cluster1) was mainly enriched in RTK–RAS and Hippo signaling pathways, while subtype B (Cluster2) showed enrichment in RTK–RAS and TP53 pathways (**Figures 8I, J**). The druggability of genes and potential drug–gene interactions in the two STAD subtypes were investigated based on mutation status using the Drug –Gene Interaction database (DGIdb). The predicted drugs primarily targeted genes in the “druggable genome” category, including ACVR2A, DST, FAT4, HMCN1, and MUC16 in subtype A (Cluster1), while the target genes in subtype B (Cluster2) included COL12A1, DMD, FAT3, FAT4, and HMCN1 (**Figures 8K, L**).

### Immune infiltration analysis of gastric cancer subtypes

**Figure 9:**
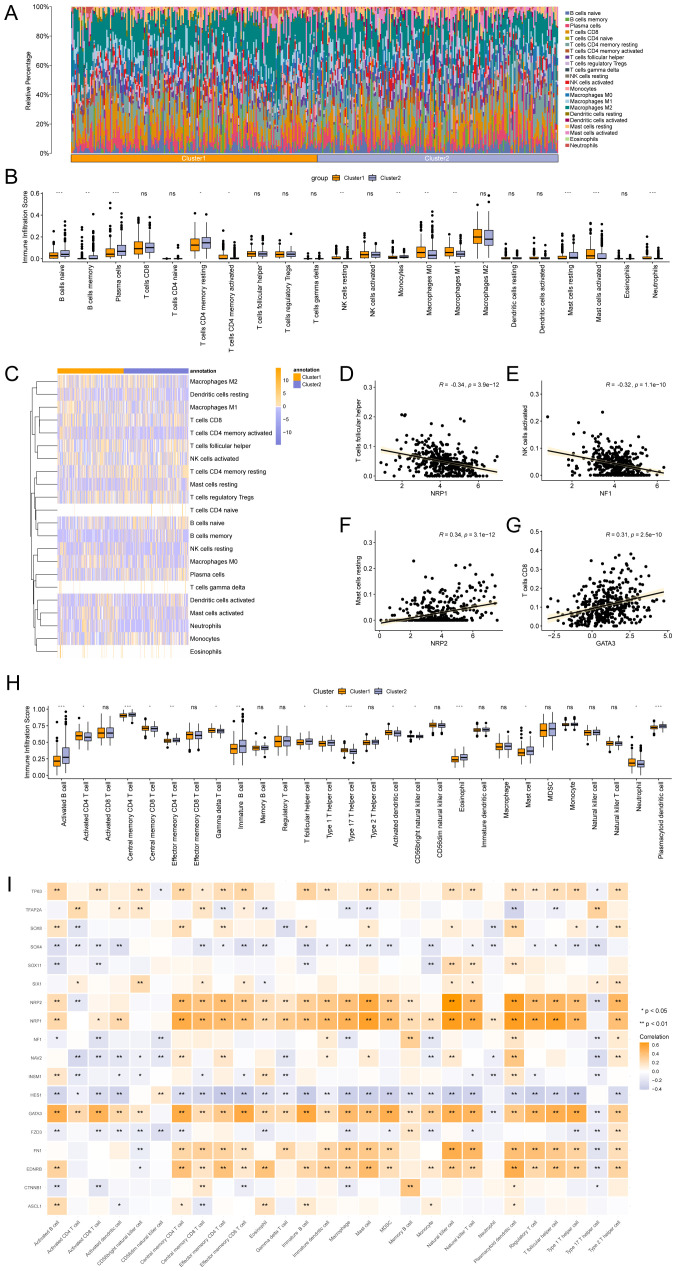
Immune infiltration analysis by CIBERSORT and ssGSEA algorithms. **(A)** Bar chart showing the proportion of immune cells. **(B)** Group comparison chart of immune cell infiltration based on the CIBERSORT algorithm. **(C)** Heatmap depicting immune infiltration. **(D–G)**. Correlation dot plots of the top genes negatively and positively correlated with immune infiltration. **(H)** Group comparison chart of immune cell infiltration based on the ssGSEA algorithm. **(I)** Heatmap of the association between Hub-ANSDR.Sig and immune cells. * represents p< 0.05; ** represents p<0.01; *** represents p<0.001.

The CIBERSORT algorithm was applied to calculate the correlation between 22 immune cells and different subtypes using the TCGA-STAD dataset. A bar chart showing the proportions of immune cells was drawn based on the immune infiltration analysis (**Figure 9A**). A group comparison diagram was used to display differences in immune cell infiltration abundance (**Figure 9B**). As shown, the expression levels of 12 immune cell types differed significantly between the two subtypes, including naive B cells, memory B cells, plasma cells, resting CD4^+^ memory T cells, activated CD4^+^ memory T cells, resting NK cells, monocytes, M0 macrophages, M1 macrophages, resting mast cells, activated mast cells, and neutrophils. Furthermore, the overall abundance of immune cell infiltration between subtypes was visualized via heat map (**Figure 9C**). The relationship between Hub-ANSDR.Sig genes and immune cell infiltration in STAD was explored through correlation analysis. The two genes with the highest positive and negative correlations were selected for display (**Figures 9D–G**). Specifically, NRP1 was significantly negatively associated with follicular helper T cell infiltration (r=-0.34), and NF1 showed a negative correlation with activated NK cell infiltration (r=-0.32). Conversely, NRP2 was positively correlated with resting mast cell infiltration (r=0.34), and GATA3 was positively correlated with CD8^+^ T cell infiltration (r=0.31).

The expression matrix from the TCGA-STAD dataset was subsequently used to calculate the infiltration abundance of 28 immune cells using the ssGSEA algorithm. Expression levels were displayed using a group comparison plot. Consequently, 16 immune cell types showed significant differences between the two subtypes, including activated B cells, activated CD4^+^ T cells, central memory CD4^+^ and CD8^+^ T cells, effector memory CD4^+^ T cells, gamma delta T cells, immature B cells, follicular helper T cells, type 1 and type 17 T helper cells, activated dendritic cells, CD56bright NK cells, eosinophils, mast cells, neutrophils, and plasmacytoid dendritic cells (**Figure 9H**). A correlation heat map revealed that NRP2, NRP1, GATA3, FN1, and EDNRB were significantly positively associated with immune infiltration, while SOX4 and HES1 showed significant negative correlations (**Figure 9I**).

### Expression difference analysis of Hub-ANSDR.Sig in GC tissues

**Figure 10:**
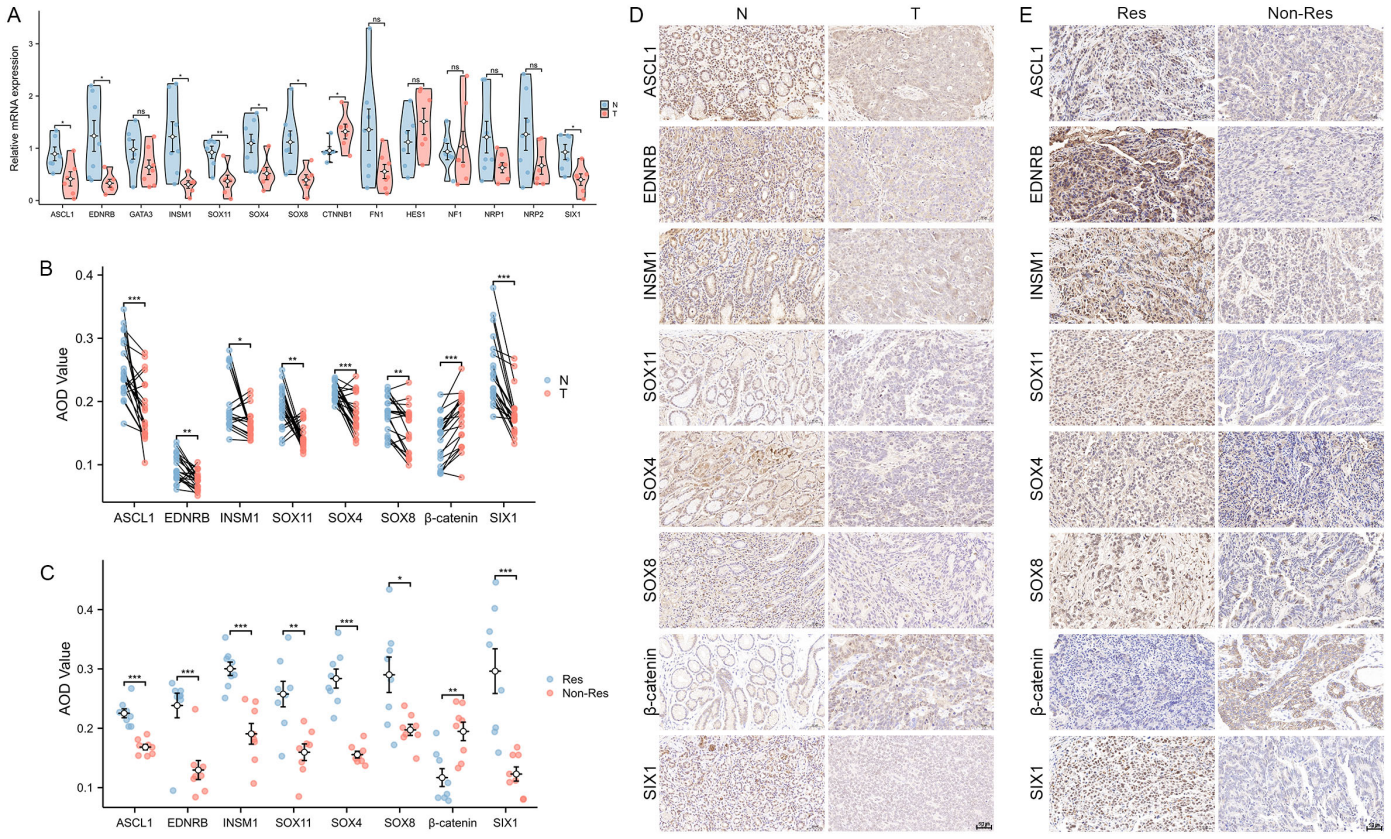
Expression of Hub-ANSDR.Sig in GC samples. **(A)** mRNA expression differences between paracancer and GC tissues (n=7 pairs, t test). **(B)** Differential protein levels in paracancer and GC tissues (n=7 pairs, Wilcoxon signed rank test). **(C)** Protein level differences in immunotherapy -sensitive and -insensitive GC patient tissues (n=16, t test). **(D)** Representative images of differential protein levels in paracancer and GC tissues. **(E)** Representative images of differential protein levels in immunotherapy -sensitive and -insensitive GC patient tissues. *p< 0.05; **p<0.01; ***p<0.001.

The mRNA levels of genes in Hub-ANSDR.Sig were measured in seven pairs of GC and corresponding paracancer tissues using RT-PCR assay. A total of eight genes showed statistically significant expression differences. Notably, lower expression of ASCL1, EDNRB, INSM1, SOX11, SOX4, SOX8, and SIX1, and relatively higher expression of CTNNB1, were observed in GC tissues compared with matched paracancer tissues (**Figure 10A**). Furthermore, similar trends in protein expression levels were authenticated by IHC analysis in the same tissue samples (**Figures 10B, D**). To explore the relationship between these eight differentially expressed genes and immunotherapy efficacy, tumor tissues from 16 GC patients—comprising eight responders and eight non-responders—were analyzed by IHC assay. As shown, ASCL1, EDNRB, INSM1, SOX11, SOX4, SOX8, and SIX1 appeared to express more abundant in Responders Group, while the level of β-catenin expression was markedly higher in the non-responder group (**Figures 10C, E**).

## Discussion

Cancer remains a primary cause of death worldwide, posing a serious threat to human health and life expectancy, second only to cardiovascular diseases (1). Immunotherapy has marked a major breakthrough in cancer treatment, designed mainly to stimulate the body’s own immune system to enhance specific antitumor immunity. Notably, ICIs are the most extensively researched and widely used treatment option in clinical practice, offering relatively stable and sustained efficacy (2, 10). However, most patients tend to exhibit poor responses, with only a small proportion deriving clinical benefit (21). Even worse, existing biomarkers show limited capacity to predict therapeutic outcomes and prognosis in advance (22). Therefore, it is of urgent necessity to devise models that can accurately forecast immunotherapy efficacy for the sake of precision medicine and expanded clinical applications.

ANSD -related genes have been universally acknowledged to be involved in various signaling pathways in cancers. Notably, ANSDR.Sig consists of a series of biomarkers including transcription factors, translocated cancer genes, cell differentiation markers, and homeodomain proteins. Abnormal expressions of these molecules may pave way for the modification of the cancer immune microenvironment. For instance, CTNNB1 has been verified as a crucial oncogene that promotes malignant features (23). Activation of GATA3 is capable of restricting the motility of luminal breast cancer cells (24). NF1 is recognized as a pivotal tumor suppressor, and its mutation can lead to a unique clinicopathologic subtype of lung adenocarcinoma (25). Moreover, the downregulation of SOX4 has been proven to promote the efficacy of CAR T cell therapy in solid tumors by reversing immune cell dysfunction (26). Therefore, ANSDR.Sig is worthy of attention as a potential tool for efficacy prediction and treatment decision-making in cancer.

Enrichment analysis highlighted the role of ANSDR.Sig in numerous biological processes, molecular functions, cellular components, and metabolic pathways. Among them, autonomic and sympathetic nervous system development were of great importance due to their potential implications for tumor diagnosis and therapy. It is well known that infiltration of certain immune cells in tumor tissues is closely associated with immunotherapy response and prognosis (27). Our immune correlation analysis indicated that ANSDR.Sig is positively related to most immune-related genes across 30 different types of cancer. Generally, ANSDR.Sig contributed to remarkable immune cell infiltration, especially in HNSC and KICH, while higher ANSDR.Sig scores were inversely associated with immune infiltration in TGCT and SARC. Furthermore, analysis of correlations between ANSDR.Sig and HALLMARK-related pathways indicated a negative effect on several biological processes linked to immunosuppression. Prediction models for immune response were next established using seven ML algorithms, and the one constructed via the RF algorithm was finally discovered to be the most predictive. More significantly, ANSDR.Sig demonstrated superior predictive capacity compared to most previously published ICI response–related gene sets. To further validate the application value of ANSDR.Sig in immunotherapy forecasting, the proportion of top-ranked genes in ANSDR.Sig and other published ICI response–associated signatures was calculated. The results showed that ANSDR.Sig had one of the highest percentages. Specifically, SOX11, TP63, FZD3, and HES1 were included in the top 20% of ranked genes and were identified as potential immune resistance markers.

Hub-ANSDR.Sig, composed of 18 genes mostly located on chromosomes 2 and 3, was subsequently derived from ANSDR.Sig using five ML algorithms. It was found to have extensive interactions with a large number of TFs and miRNAs. Kaplan–Meier curve analysis confirmed that a higher RiskScore based on Hub-ANSDR.Sig was significantly associated with poorer OS. This trend was also validated in LGG, MESO, and STAD cohorts. As widely recognized, immune infiltration signatures represent antitumor activity and serve as reliable indicators for prognosis and treatment prediction in multiple cancers (28). Our panoramic analysis revealed that Hub-ANSDR.Sig was positively associated with resting CD4 memory T cells but negatively correlated with CD8 T cells and Tregs. MSI status is generally acknowledged to be predictive of chemotherapy response and clinical outcome (29). Hub-ANSDR.Sig showed the strongest positive correlation with MSI in COAD, while the most negative association was discovered in HNSC. More specifically, SOX8 was the top negatively correlating gene with MSI in KICH, while TFAP2A showed the strongest positive correlation in COAD. These findings suggest that Hub-ANSDR.Sig holds promise as a more accurate predictive tool for guiding immunotherapy strategies.

Gastric cancer is widely recognized as a prevalent digestive malignancy threatening human health around the world. Although immunotherapy shows significant efficacy in a subset of patients, a considerable proportion of GC patients, particularly those with excluded or desert immune phenotypes, derive little benefit from current immune-based interventions (30). Therefore, identification of GC subtypes based on specific gene signatures is urgently needed for more precise efficacy prediction and clinical decision-making (31). In our study, two GC subtypes were distinguished by clustering based on the expression of Hub-ANSDR.Sig using the TCGA-STAD dataset, and a notable difference in dysfunction, TMB, and MSI was observed between the two groups. Furthermore, TTN and TP53 were identified as the most frequently mutated genes in subtype A and subtype B, respectively, and were associated with distinct functional pathway alterations. As precision medicine advances, research on novel gene features aims to support the development of innovative therapies (32). During our study, the molecular mutation in the two subtypes were leveraged to explore potential drugs targeting different gene profiles, reinforcing the translational potential of Hub-ANSDR.Sig in drug development. GC is well known for its heterogeneity, largely due to variations in its immune microenvironment (33). We employed two algorithms to analyze immune cell infiltration in the two GC subtypes and identified immune cells with differential abundance between subtypes. Several genes in Hub-ANSDR.Sig were further verified to influence specific immune infiltration patterns. Additionally, ASCL1, EDNRB, INSM1, SOX11, SOX4, SOX8, and SIX1 were found to be more enriched in GC tissues from immunotherapy -sensitive patients, whereas β-catenin expression was significantly higher in immune-resistant tissues. Our results highlighted the importance of exploring the role of Hub-ANSDR.Sig in shaping the immune microenvironment, which might lead to innovative treatment strategies in GC. It is universally acknowledged that intercellular communication forms a complex regulatory network that governs therapeutic responses (34). Based on our cell communication analysis, Hub-ANSDR.Sig was shown to participate in interactions between immune and non-immune cell types, affect anticancer immune responses, and modulate receptor–ligand expression and interaction intensity. These results highlight the underlying value of Hub-ANSDR.Sig in GC development and treatment outcome. The autonomic nervous system plays a key role in maintaining epithelial homeostasis and regulating gastric tumorigenesis through mechanisms involving neurotransmission, neuroimmune modulation, and inflammatory signaling (35). Our pseudotime analysis revealed that cytotoxic T cells, mast cells, and dendritic cells are likely to occupy early positions in the developmental trajectory. These cells are known to strongly influence tumor burden and antitumor immune responses in GC (36–38). Gastric epithelial cells, myofibroblasts, and fibroblasts appeared at later stages, and the crosstalk among these cell types is known to affect the GC immune microenvironment (39). We examined the dynamic expression and kinetic behavior of Hub-ANSDR.Sig during this trajectory. Specifically, NRP2 and GATA3 were preferentially expressed in one group, followed by ASCL1 and INSM1 in another. Most of these genes are essential regulators of malignant phenotypes in GC (40, 41). Afterwards, the expression profiles of Hub-ANSDR.Sig were examined in paired GC patient samples. Eight genes were differentially expressed at both the mRNA and protein levels, with ASCL1, EDNRB, INSM1, SOX11, SOX4, SOX8, and SIX1 showing reduced expression in tumor tissues, and CTNNB1 showing increased expression compared with para-cancer tissues.

## Conclusions

To sum up, our findings reveal the promising application potential of a prediction tool based on ANSD-related signature, which showed stable and efficient forecasting ability for immunotherapy efficacy and prognosis in cancer. Further studies may contribute to improved clinical decision-making and even facilitate the discovery of novel therapeutic targets, ultimately benefiting more patients. However, we acknowledge some limitations in the present study. First, due to the integration of numerous publicly available datasets, the impact of batch effects could not be fully eliminated. Second, experimental validation at the functional level is currently lacking. In-depth exploration of the regulatory network and mechanisms underlying Hub-ANSDR.Sig remains to be further elucidated. In future work, we will conduct mechanistic studies using cellular and animal models to reinforce our findings. We also plan to perform systematic genetic interventions targeting the identified genes, in combination with high-throughput omics approaches, to further uncover their molecular mechanisms in regulating the tumor immune microenvironment. Finally, due to limitations in available data resources and research scope, multi-omics datasets such as ceRNA networks or chromatin accessibility profiles have not yet been integrated, leaving the regulatory network incomplete. Furthermore, partial incompatibility among certain publicly available datasets—particularly in terms of expression matrix formatting and the completeness of clinical information—currently prevents a comprehensive comparison between the ANSDR.Sig-based model and other mainstream predictive tools across all cohorts. Nonetheless, we firmly believe that with the continued accumulation of high-quality, multi-center immunotherapy cohorts and more comprehensive expression and clinical data, larger-scale clinical validation studies will soon be feasible to further validate the clinical applicability of the proposed model.

## Data Availability

The original contributions presented in the study are included in the article/**Supplementary Material**. Further inquiries can be directed to the corresponding authors.
