# Supplementary material for: Autonomic nervous system development-related signature as a novel predictive biomarker for immunotherapy in pan-cancers
Source: Front Immunol. 2025 Jul 23;16:1611890. doi: 10.3389/fimmu.2025.1611890 (PMC12325192; doi:10.3389/fimmu.2025.1611890)
Supplement: Supplementary file 1 [file DataSheet1.docx]

**Availability of data and material**

The autonomic nervous system related genes were collected through the Molecular Signatures Database (MSigDB) by taking “Autonomic Nervous System” as key words. The GOBP_AUTONOMIC_NERVOUS_SYSTEM_DEVELOPMENT data set contains a total of 46 genes related to Autonomic Nervous System (https://www.gsea-msigdb.org/gsea/msigdb/human/geneset/GOBP_AUTONOMIC_NERVOUS_SYSTEM_DEVELOPMENT.html).

Two datasets with clear tumor immunotherapy efficacy, including SKCM related data set GSE115978 and BCC related data set GSE123813 were downloaded from the GEO database (https://www.ncbi.nlm.nih.gov/geo/) through R package GEOquery.

Thirty-four scRNA-Seq datasets contained stromal or immune cells were collected from the TISCH database (http://tisch.comp-genomics.org/), comprising 345 patients and 663760 cells. The datasets cover 17 cancer types including BCC, SKCM, Breast Cancer (BRCA), Cholangiocarcinoma (CHOL), colorectal Cancer (CRC), Glioma (GBM), HNSC, Liver Hepatocellular Cancer (LIHC), Medulloblastoma (MB), Merkel Cell Carcinoma (MCC), Multiple Myeloma (MM), Neuroendocrine Tumor, Non-small Cell Lung Cancer (NSCLC), Ovarian Serous Cystadenocarcinoma (OV), Pancreatic Adenocarcinoma (PAAD), STAD and Uveal Melanoma (UVM).

The pan-cancer transcriptome data set of The Cancer Genome Atlas (TCGA) was downloaded from the UCSC Xena database (http://xena.ucsc.edu). TMB and MSI data were downloaded from the cBioPortal database (http://cbioportal.org). The relevant clinicopathological information of 30 different cancer types was downloaded through R package TCGAbiolink. The survival data all met the following criteria containing mRNA expression and clinical data. All patients had completed standardized diagnosis and treatment, who survived for more than 30 days.

Eight ICI related RNA-Seq datasets consisting of four SKCM related datasets (Hugo 2016 [1], Liu 2019 [2], Gide 2019 [3], Riaz 2017 [4]), two Urothelial Carcinoma (UC) related datasets (Mariathasan 2018 [5], Snyder 2017 [6]), one GBM related data set (Zhao 2019 [7]), and one Renal Cell Carcinoma (RCC) related data set (Braun 2020 [8]). The data set Hugo 2016 of SKCM contained 27 pre-treated tumor samples from 26 patients, and the data set Zhao 2019 GBM included 34 pre-treated tumor samples from 17 patients. For these two data groups, one tumor sample was randomly selected for each patient. Seven pan-cancer signatures (IPRES.Sig [1], INFG.Sig [9], T.cell.inflamed.Sig [9], PDL1.Sig [10], LRRC15.CAF.Sig [11], NLRP3.Sig [12], Cytotoxic.Sig [13]) and four SKCM specific signatures (CRMA.Sig [14], ImmuCells.Sig [15], IMS.Sig [16], TRS.Sig [17]) gene lists were collected . The codes and algorithms of the above 11 signatures were derived from the original studies, such as ssGSEA of NLRP3.Sig and cancer classification of ImmuneCell.Sig.

**References**

1. Hugo W, Zaretsky JM, Sun L, Song C, Moreno BH, Hu-Lieskovan S, et al. Genomic and transcriptomic features of response to anti-PD-1 therapy in metastatic melanoma. Cell. (2016) 165:35–44. doi: 10.1016/j.cell.2016.02.065

2. Liu D, Schilling B, Liu D, Sucker A, Livingstone E, Jerby-Amon L, et al. Integrative molecular and clinical modeling of clinical outcomes to PD1 blockade in patients with metastatic melanoma. Nat Med. (2019) 25:1916–27. doi: 10.1038/s41591-019-0654-5

3. Gide TN, Quek C, Menzies AM, Tasker AT, Shang P, Holst J, et al. Distinct immune cell populations define response to anti-PD-1 monotherapy and anti-PD-1/anti-CTLA-4 combined therapy. Cancer Cell. (2019) 35:238–255.e6. doi: 10.1016/j.ccell.2019.01.003

4. Riaz N, Havel JJ, Makarov V, Desrichard A, Urba WJ, Sims JS, et al. Tumor and microenvironment evolution during immunotherapy with nivolumab. Cell. (2017) 171:934–949.e16. doi: 10.1016/j.cell.2017.09.028

5. Mariathasan S, Turley SJ, Nickles D, Castiglioni A, Yuen K, Wang Y, et al. TGFb attenuates tumour response to PD-L1 blockade by contributing to exclusion of T cells. Nature. (2018) 554:544–8. doi: 10.1038/nature25501

6. Snyder A, Nathanson T, Funt SA, Ahuja A, Buros Novik J, Hellmann MD, et al. Contribution of systemic and somatic factors to clinical response and resistance to PDL1 blockade in urothelial cancer: An exploratory multi-omic analysis. PloS Med. (2017) 14:e1002309. doi: 10.1371/journal.pmed.1002309

7. Zhao J, Chen AX, Gartrell RD, Silverman AM, Aparicio L, Chu T, et al. Immune and genomic correlates of response to anti-PD-1 immunotherapy in glioblastoma. Nat Med. (2019) 25:462–9. doi: 10.1038/s41591-019-0349-y

8. Braun DA, Hou Y, Bakouny Z, Ficial M, Sant’ Angelo M, Forman J, et al. Interplay of somatic alterations and immune infiltration modulates response to PD-1 blockade in advanced clear cell renal cell carcinoma. Nat Med. (2020) 26:909–18. doi: 10.1038/s41591­020-0839-y

9. Ayers M, Lunceford J, Nebozhyn M, Murphy E, Loboda A, Kaufman DR, et al. IFN-g-related mRNA profile predicts clinical response to PD-1 blockade. J Clin Invest. (2017) 127:2930–40. doi: 10.1172/JCI91190

10. Topalian SL, Hodi FS, Brahmer JR, et al. Safety, activity, and immune correlates of anti-PD-1 antibody in cancer. N Engl J Med. (2012) 366:2443–54. doi: 10.1056/NEJMoa1200690

11. Dominguez CX, Müller S, Keerthivasan S, et al. Single-cell RNA sequencing reveals stromal evolution into LRRC15+ Myofibroblasts as a determinant of patient response to cancer immunotherapy. Cancer Discov. (2020) 10:232–53. doi: 10.1158/2159-8290.CD-19-0644

12. Ju M, Bi J, Wei Q, et al. Pan-cancer analysis of NLRP3 inflammasome with potential implications in prognosis and immunotherapy in human cancer. Brief Bioinform. (2021) 22:bbaa345. doi: 10.1093/bib/bbaa345

13. Rooney MS, Shukla SA, Wu CJ, Getz G, Hacohen N. Molecular and genetic properties of tumors associated with local immune cytolytic activity. Cell. (2015) 160:48–61. doi: 10.1016/j.cell.2014.12.033

14. Shukla SA, Bachireddy P, Schilling B, et al. Cancer-germline antigen expression discriminates clinical outcome to CTLA-4 blockade. Cell. (2018) 173:624–633.e8. doi: 10.1016/j.cell.2018.03.026

15. Xiong D, Wang Y, You M. A gene expression signature of TREM2hi macrophages and gd T cells predicts immunotherapy response. Nat Commun. (2020) 11:5084. doi: 10.1038/s41467-020-18546-x

16. Cui C, Xu C, Yang W, et al. Ratio of the interferon-p signature to the immunosuppression signature predicts anti-PD-1 therapy response in melanoma. NPJ Genom Med. (2021) 6:7. doi: 10.1038/s41525-021-00169-w

17. Yan M, Hu J, Ping Y, et al. Single-cell transcriptomic analysis reveals a tumorreactive T cell signature associated with clinical outcome and immunotherapy response in melanoma. Front Immunol. (2021) 12:758288. doi: 10.3389/fimmu.2021.758288
