# Supplementary material for: Autonomic nervous system development-related signature as a novel predictive biomarker for immunotherapy in pan-cancers
Source: Front Immunol. 2025 Jul 23;16:1611890. doi: 10.3389/fimmu.2025.1611890 (PMC12325192; doi:10.3389/fimmu.2025.1611890)
Supplement: Supplementary file 2 [file Table1.docx]

| ONTOLOGY | ID | Description | GeneRatio | BgRatio | pvalue | p.adjust | qvalue |
| --- | --- | --- | --- | --- | --- | --- | --- |
| BP | GO:0048483 | autonomic nervous system development | 20/20 | 48/18800 | 1.35E-54 | 2.19E-51 | 8.16E-52 |
| BP | GO:0048485 | sympathetic nervous system development | 12/20 | 22/18800 | 2.00E-32 | 1.62E-29 | 6.03E-30 |
| BP | GO:0001655 | urogenital system development | 13/20 | 352/18800 | 1.92E-18 | 1.04E-15 | 3.86E-16 |
| BP | GO:0061548 | ganglion development | 7/20 | 17/18800 | 9.11E-18 | 3.68E-15 | 1.37E-15 |
| BP | GO:0001822 | kidney development | 12/20 | 303/18800 | 2.78E-17 | 8.98E-15 | 3.35E-15 |
| CC | GO:0005667 | transcription regulator complex | 6/20 | 483/19594 | 6.28E-06 | 5.84E-04 | 3.97E-04 |
| CC | GO:0002116 | semaphorin receptor complex | 2/20 | 11/19594 | 5.41E-05 | 2.52E-03 | 1.71E-03 |
| CC | GO:0016328 | lateral plasma membrane | 2/20 | 64/19594 | 1.92E-03 | 4.18E-02 | 2.84E-02 |
| CC | GO:0045211 | postsynaptic membrane | 3/20 | 271/19594 | 2.51E-03 | 4.18E-02 | 2.84E-02 |
| CC | GO:0048786 | presynaptic active zone | 2/20 | 75/19594 | 2.63E-03 | 4.18E-02 | 2.84E-02 |
| MF | GO:0001228 | DNA-binding transcription activator activity, RNA polymerase II-specific | 8/20 | 462/18410 | 1.43E-08 | 6.28E-07 | 3.06E-07 |
| MF | GO:0001216 | DNA-binding transcription activator activity | 8/20 | 466/18410 | 1.53E-08 | 6.28E-07 | 3.06E-07 |
| MF | GO:0001221 | transcription coregulator binding | 4/20 | 108/18410 | 5.05E-06 | 1.38E-04 | 6.73E-05 |
| MF | GO:0140297 | DNA-binding transcription factor binding | 6/20 | 470/18410 | 7.67E-06 | 1.57E-04 | 7.67E-05 |
| MF | GO:0001227 | DNA-binding transcription repressor activity, RNA polymerase II-specific | 5/20 | 321/18410 | 1.95E-05 | 2.43E-04 | 1.18E-04 |
| KEGG | hsa04916 | Melanogenesis | 3/11 | 101/8164 | 2.82E-04 | 1.64E-02 | 1.22E-02 |
| KEGG | hsa05165 | Human papillomavirus infection | 4/11 | 331/8164 | 6.98E-04 | 1.64E-02 | 1.22E-02 |
| KEGG | hsa05224 | Breast cancer | 3/11 | 147/8164 | 8.49E-04 | 1.64E-02 | 1.22E-02 |
| KEGG | hsa05217 | Basal cell carcinoma | 2/11 | 63/8164 | 3.08E-03 | 3.58E-02 | 2.66E-02 |
| KEGG | hsa05100 | Bacterial invasion of epithelial cells | 2/11 | 77/8164 | 4.57E-03 | 4.42E-02 | 3.29E-02 |
