# Supplementary material for: Autonomic nervous system development-related signature as a novel predictive biomarker for immunotherapy in pan-cancers
Source: Front Immunol. 2025 Jul 23;16:1611890. doi: 10.3389/fimmu.2025.1611890 (PMC12325192; doi:10.3389/fimmu.2025.1611890)
Supplement: Supplementary file 5 [file Table4.docx]

| Target | Primer sequence（5’-3’) | Size（bp） |
| --- | --- | --- |
| H-GAPDH-S | GGAAGCTTGTCATCAATGGAAATC | 168 |
| H-GAPDH-A | TGATGACCCTTTTGGCTCCC |  |
| H-ASCL1-S | GCCAACAAGAAGATGAGTAAGGTG | 133 |
| H-ASCL1-A | AGATGGTGGGCGACAGGA |  |
| H-EDNRB-S | GCTTGCTTCATCCCGTTCAGA | 239 |
| H-EDNRB-A | GAGGGCAAAGACAAGGACCAG |  |
| H-GATA3-S | CAAGCTTCACAATATTAACAGACCC | 144 |
| H-GATA3-A | GGGTTAAACGAGCTGTTCTTGG |  |
| H-INSM1-S | GCCATCACTGCGCCAAGA | 130 |
| H-INSM1-A | GGGTACAAGGCCAGTAGGTCC |  |
| H-SOX11-S | CCCTGTCGCTGGTGGATAAG | 82 |
| H-SOX11-A | GTGCAGTAGTCGGGGAACTC |  |
| H-SOX4-S | CTCAAGCACATGGCTGACTACC | 135 |
| H-SOX4-A | ACTGCCACCGACCTTGTCTC |  |
| H-SOX8-S | GCGCCAGAACATCGACTTCA | 106 |
| H-SOX8-A | GGGCAGGTACTGGTCGAACT |  |
| H-CTNNB1-S | GTGCTGAAGGTGCTATCTGTCTG | 142 |
| H-CTNNB1-A | CTGAAAGATTCCTGAGAGTCCAAAG |  |
| H-FN1-S | GGAGAGTGGAAGTGTGAGAGGC | 276 |
| H-FN1-A | TCCATTTGAGTTGCCACCGT |  |
| H-HES1-S | ACACGACACCGGATAAACCAAA | 152 |
| H-HES1-A | ATGCCGCGAGCTATCTTTCTT |  |
| H-NF1-S | CCACAGACTGATATGGCTGAATG | 114 |
| H-NF1-A | GATAAGGAGAATGATTTGTAGTGGC |  |
| H-NRP1-S | CCCTCACATTGGGCGTTACTG | 185 |
| H-NRP1-A | ATTCCATGCCCAGAGCTTCC |  |
| H-NRP2-S | CAGGGAAACACAGAATGGCTACT | 119 |
| H-NRP2-A | CGTTGTTGGCTTGAAATACCTT |  |
| H-SIX1-S | TGCCGTCGTTTGGCTTTA | 132 |
| H-SIX1-A | TCTCGTTCTTGTGCAGGTGG |  |
